# Predictors of all-cause 1-year mortality in myocardial infarction patients

**DOI:** 10.1097/MD.0000000000021288

**Published:** 2020-07-17

**Authors:** Qing Ye, Jie Zhang, Likun Ma

**Affiliations:** Department of Cardiology, The First Affiliated Hospital of USTC, Division of Life Sciences and Medicine, University of Science and Technology of China, Hefei, Anhui 230036, PR China.

**Keywords:** all-cause 1-year mortality, left ventricular ejection fraction, logistic regression, myocardial infarction

## Abstract

Compared with the general population, myocardial infarction (MI) survivors have a higher risk of mortality in the first year after the index event.

The aim of this study was to determine the associations between variables obtained during the index admission and 1-year all-cause mortality on follow-up.

A cohort of 296 patients was enrolled in the study, with a median age of 63.8 ± 12.68 years. All patients received a coronary angiography and stent implantation by percutaneous coronary intervention. Each variable was tested for association with all-cause mortality, using chi-square tests for categorical and binary variables and *t* tests for continuous variables. The relative prognostic power of each significant variable was further evaluated by logistic regression before and after adjustment for differences in baseline characteristics.

Patients who were deceased after 1-year of MI had significantly higher mean age, increased prevalence of diabetes, and elevated heart rate as compared to those who were surviving. Univariate analysis indicated that patient mortality within 1-year of MI was strongly correlated with higher rates of pump failure on admission (*P* < .0001), bleeding complications (*P* = .02), the severity of coronary artery disease measured by Gensini score (*P* = .04), and decreased left ventricular ejection fraction (LVEF) (*P* < .0001). After adjustment of baseline variables, only pump failure (*P* = .006) and reduced LVEF (*P* < .0001) were independently associated with 1-year mortality.

Our study shows that LVEF dysfunction and pump failure are independent predictors of 1-year all-cause post-MI mortality, while the severity of coronary artery disease and bleeding did not qualify as independent predictors. Also, age, history of diabetes, and elevated heart rate may be used as markers for increased mortality rates.

## 1 Introduction

Long-term survival after myocardial infarction (MI) has improved over the last 3 decades in developed countries.^[1–8]^ However, approximately 20% of patients experiencing an acute MI die within 1 year of the event, with over half the first-year mortality occurring after 30 days of MI.^[2]^ To accurately predict survival after MI, 1 has to take into account multiple organ systems and comorbidities that may interact with heart disease and affect overall mortality.^[3]^ The list of variables affecting post-MI mortality rates includes a gender,^[6]^ age,^[7]^ smoking, history of diabetes,^[4]^ renal failure,^[5]^ hypertension, peripheral artery disease, stroke, chronic obstructive pulmonary disease, chronic liver disease, and cancer.^[2,8]^ In addition to these risk factors, numerous reports also show an association between increased annual mortality after MI and additional clinical parameters, such as elevated resting heart rate,^[9]^ diagnosed pump failure on admission,^[10]^ left ventricular ejection fraction (LVEF) dysfunction,^[11]^ bleeding complications,^[12,13]^ and a history of obstructive coronary artery disease.^[8]^

The goal of this paper is to evaluate if any of these parameters could be independent predictors of death after 1 year of MI. Identification of these variables would help to develop and validate statistical models that can be used to determine 1-year mortality after an acute MI, and ensure intensive follow-up and risk factor modification.

## 2 Methods

### 2.1 Study design

This institutional ethical committee approved retrospective study (approval no. 2019KY11) included 330 patients with a diagnosis of MI admitted to the First Affiliated Hospital of USTC between January 1, 2018 and December 30, 2019. Since data was obtained from de-identified medical records and involved no patient interaction, informed consent was waived for the purpose of this study.

All included patients had received a coronary angiography and stent implantation by percutaneous coronary intervention during their admission. The following data were recorded for all patients at baseline admission: demographic details, medical history, cardiovascular clinical details like pump failure, cardiac shock, malignant arrhythmia, recurrent MI, apoplexy, and bleeding based on the Bleeding Academic Research Consortium (BARC) classification. Further clinical data recorded included the patient's angiography details like number of diseased vessels, specification of the occluded artery; intraoperative medications administered (heparin, bivalirudin, tirofiban); echocardiography data (LVEF, left ventricular systolic function, left ventricular diastolic function); as well as the Gensini risk score for severity of coronary artery disease.

After a follow-up period of 1 year, we sourced data on recurrent MI, apoplexy, heart failure, BARC bleeding classification, and mortality from the patient records. Fifteen patients that were lost to follow-up and 19 patients with missing data on all-cause mortality were excluded from the study, leaving a patient cohort of 296 valid cases.

### 2.2 Statistical analysis

Baseline variables were tested for association with all-cause mortality, using chi-square tests for categorical and binary variables and *t* tests for continuous variables. All data were checked for quality, including reasonability and consistency of units of measure. All variables that showed a significant association with all-cause mortality were further tested using logistic regression to assess whether associations remained significant after adjusting for age and all significant patient history variables. Age was included in all models as it was strongly associated with all-cause mortality consistently for each variable tested and could otherwise confound the effects of patient history variables.

Statistical analyses were performed using R Software Version 3.5.3 (R Core Team, 2019). Continuous data was presented as mean ± standard deviation and categorical data was presented as counts (% of total). For comparisons that were significant, multiple variable logistic regression was used to calculate odds ratios (ORs) with 95% confidence intervals (CIs) after adjusting for demographic and patient history variables. For all tests, *P*-values <.05 were considered statistically significant.

## 3 Results

### 3.1 Baseline characteristics

Baseline characteristics for the study population are presented in Table 1. Two hundred ninety-six patients were included in the study, with a median age of 63.8 ± 12.68 years, and a median weight of 69.79 ± 13.6 kg. The majority of the patients were males (75.1%). One-year survival of the cohort was 91.2% (270 patients). To compare baseline differences, the patients were divided into 2 groups, alive and deceased.

**Table 1:**
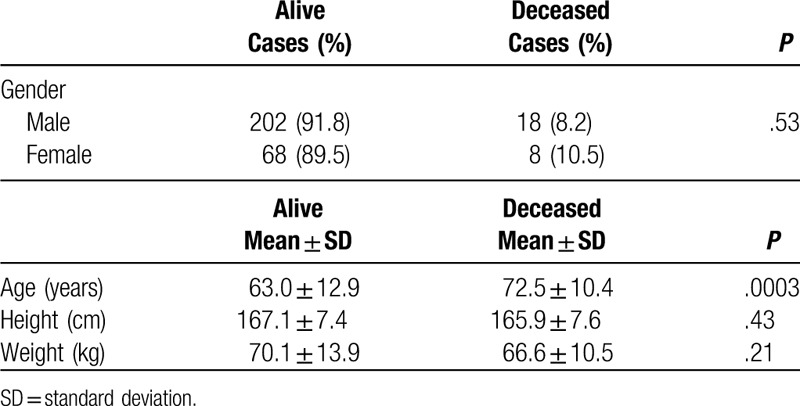
Demographic variables.

Patients in the deceased group were significantly older than in the surviving group, with a median age of 72.5 ± 10.4 as opposed to 63 ± 12.9 in the surviving group (*P* = .0003). As summarized in Table 2, there were no differences between the 2 groups for history of cerebrovascular disease (CVD) or hypertension as well as baseline systolic and diastolic blood pressures. However, patients in the deceased group had significantly higher prevalence of diabetes (*P* = .01) and elevated heart rate on admission (*P* = .02). The deceased group had significantly higher incidence of pump failure (*P* < .0001) and BARC defined bleeding complications, ranging from type 1 to type 5b (*P* = .01).

**Table 2:**
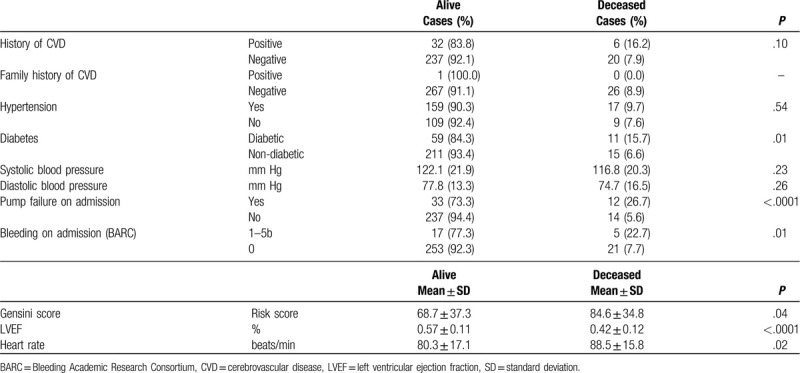
Medical history and clinical variables.

The Gensini risk score in the deceased group was significantly higher as compared to the surviving group (84.6 ± 34.8 vs 68.7 ± 37.3) (*P* = .05). Also LVEF was significantly reduced in the deceased group as compared to the surviving group (*P* < .0001).

### 3.2 Prognostic factors and predictors of 1-year mortality

We next performed a multivariate logistic regression analysis to identify possible predictors of 1-year mortality after MI. Unadjusted models are summarized in Table 3. Our results indicate strong association of 1-year all-cause mortality with pump failure [OR 6.16 (95% CI 2.62–14.44), *P* < .0001], LVEF (%) of <0.5 [OR 0.89 (95% CI 0.85–0.93), *P* < .0001], and severity of coronary artery disease as indicated by the Gensini risk score [OR 3.54 (95% CI 1.19–10.56), *P* < .05], and bleeding (1–5b BARC classification) [OR 1.01 (95% CI 1.00–1.02), *P* < .05].

**Table 3:**
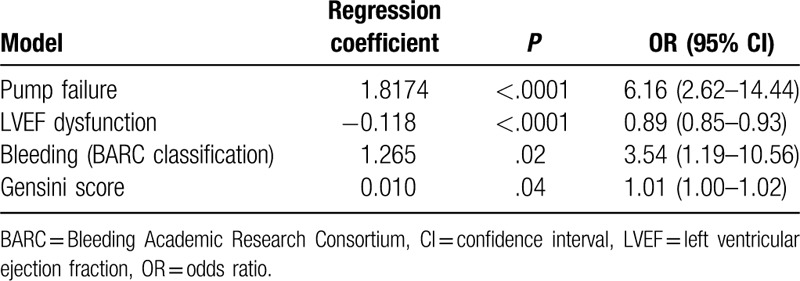
Unadjusted risk factors of all-cause post-MI mortality after 1-year of follow-up.

After adjustment for age, medical history parameters, diabetes, and heart rate, only pump failure and LVEF parameters remained statistically significant independent predictors of 1-year post-MI mortality, while the severity of coronary artery disease measured by the Gensini score and bleeding were not found to be statistically significant predictors of mortality (Table 4).

**Table 4:**
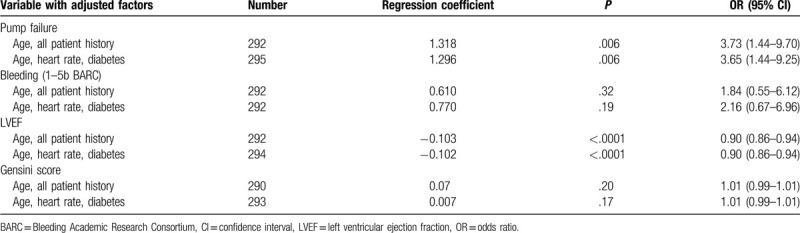
Predictors of 1-year all-cause post-MI mortality, adjusted.

## 4 Discussion

Our data suggest that the presence of pump failure and LVEF dysfunction on admission are strong independent predictors of 1-year mortality following MI. We also confirm age, history of diabetes, and increased heart rate as significant risk factors of increased post-MI mortality.

Our findings are consistent with the results of previous investigators. Numerous studies have shown that the risk of cardiovascular events and patient mortality is highest in the first year following MI.^[1,14,15]^ Patients with accompanying conditions such as hypertension, diabetes, peripheral artery disease, or history of stroke are known to have significantly higher rates of mortality.^[16]^ Accurate prediction of post-MI mortality, therefore, has to take into account multiple variables and comorbidities that impact heart disease.^[2]^ In this study we identified baseline parameters such as age, diabetes, and heart rate on admission to strongly correlate with the survival of patients after 1 year of MI. Age is one of the most significant prognostic parameters of post-MI death, partially due to the high risk of vascular events after MI in elderly patients.^[17]^ Our data confirms that there is a strong association between the risk of death and age, with almost a 10-year difference in the mean age of patients who did not survive the first-year post-MI compared to the surviving group (75.5 years versus 63 years).

Our results also indicate that 1-year mortality post-MI is significantly higher in patients with a history of diabetes. Our observations are in agreement with studies that have shown the impact of diabetes on mortality rates after MI.^[4,18]^ Population studies have shown that the post-MI mortality rates are doubled in patients with diabetes, and are equivalent to the effect of 15 years of ageing.^[19]^

Elevated heart rate at the time of hospital admission for acute MI is known to be an independent predictor of short- and long-term mortality. It is also a reliable measure of autonomic tone and physiologic stress.^[20–22]^ In our study, increased heart rate was associated with increased chances of mortality after 1-year post-MI. Taken together, our results further confirm that the survivors of MI are at higher risk of mortality 1 year after the event when risk factors such as old age, diabetes, and elevated heart rate are present. These variables could serve as simple markers that can be easily used in risk assessment of patients.

In addition to baseline variables, we looked at several clinical parameters collected on admission, such as severity of coronary artery disease, bleeding complications, LVEF dysfunction, and pump failure as potential predictors of all-cause mortality in our cohort. In our study, when no other variables were taken into account, bleeding (1–5b, BARC classification), lowered LVEF, Gensini score indicating obstructive coronary artery disease, and pump failure on admission were associated with statistically significant increase in post-MI death rates. However, after adjusting for baseline variables, such as age, patient medical history, heart rate, and diabetes, only prognostic values of pump failure and LVEF dysfunction persisted, while bleeding and high Gensini score did not qualify as independent predictors for 1-year mortality after MI.

Pump failure and LVEF dysfunction are considered among the leading causes of mortality in patients with heart failure. Narang et al^[23]^ reported that circulatory failure was the most frequently reported mode of death in chronic heart failure, accounting for up to 42% of all deaths, and noted that pre-existing chronic heart failure significantly impacted survival of MI patients. Pump failure is becoming a leading cause of mortality in patients with newly diagnosed or severe heart failure, and patients with heart failure associated with Chagas’ disease.^[24]^ In our study, pump failure on admission was also a strong independent predictor of 1-year all-cause mortality after MI, with its predictive value unaffected by correction for any baseline variables. This finding supports previous reports that over 70% of patients who survived MI, died of uncontrolled pump failure during the follow-up period.^[25]^

Low LVEF values were a significant independent predictor of 1-year death in our study. This is in agreement with earlier reports that LVEF of <40% is an independent predictor of mortality after MI.^[26]^ Our data further strengthens the importance of addressing timely treatment of heart failure with reduced LVEF, as implantable cardioverter-defibrillator and pharmacologic interventions with agents such as angiotensin-converting enzyme inhibitors and β-blockers may significantly increase survival rate of MI patients.^[27]^

The study results should be interpreted keeping in mind the study limitations. Firstly, the sample size of the study was not very high. Secondly, due to limited follow-up, our study presents data of only 1-year mortality. With the present information, it is not known if the analyzed variables also predict long-term mortality after MI.

To conclude, in our current analysis, we explored the impact of older age, comorbidities such as diabetes and coronary artery disease, and clinical parameters on admission on all-cause mortality 1 year after MI. Pump failure, severity of coronary artery disease, LVEF dysfunction or bleeding complications may serve as reliable preliminary predictors of post-MI mortality, but only pump failure on admission and lower LVEF may be considered independent predictors. Age, history of diabetes, and elevated heart rate were associated with increased mortality rates in our cohort, and could be used as markers for risk assessment.

## Author contributions

QY and LM conceived and designed the study. QY and JZ collected and analyzed the data. QY was involved in the writing of the manuscript. All authors have read and approved the final manuscript.

**Conceptualization:** Qing Ye, Jie Zhang.

**Data curation:** Qing Ye, Jie Zhang, Likun Ma.

**Formal analysis:** Qing Ye, Jie Zhang.

**Funding acquisition:** Likun Ma.

**Investigation:** Likun Ma.

**Methodology:** Qing Ye, Jie Zhang.

**Project administration:** Likun Ma.

**Resources:** Likun Ma.

**Software:** Qing Ye, Jie Zhang.

**Supervision:** Likun Ma.

**Validation:** Qing Ye, Jie Zhang, Likun Ma.

**Visualization:** Qing Ye, Jie Zhang, Likun Ma.

**Writing – original draft:** Qing Ye.

**Writing – review & editing:** Likun Ma.
